# Immunobiology of Newcastle Disease Virus and Its Use for Prophylactic Vaccination in Poultry and as Adjuvant for Therapeutic Vaccination in Cancer Patients

**DOI:** 10.3390/ijms18051103

**Published:** 2017-05-20

**Authors:** Volker Schirrmacher

**Affiliations:** Immunological and Oncological Center Cologne (IOZK), Hohenstaufenring 30-32, D-50674 Köln, Germany; V.Schirrmacher@web.de; Tel.: +49-221-4203-9925; Fax: +49-221-4203-9926

**Keywords:** cancer vaccine, dsRNA, EBOV, HN, ICD, interferon, immune escape, NDV, RIG-I, V protein

## Abstract

Newcastle disease (ND) is one of the most important diseases of poultry worldwide. In the last decades, molecular research has gained a lot of new information about its causative agent, *newcastle disease virus* (NDV). In poultry industry, certain strains of NDV have been used for preventive vaccination for more than 60 years. NDV has also been applied to cancer patients with beneficial effects for about 50 years, but this is less well known. The molecular basis for these differential effects of NDV in birds and man have been elucidated in the last decades and are explained in this review. The anti-neoplastic and immune-stimulatory properties in non-permissive hosts such as mouse and man have to do with the strong type I interferon responses induced in these foreign species. Additionally, NDV has the potential to break various types of tumor resistances and also to affect liver fibrosis. A main section is devoted to the benefits of clinical application of NDV and NDV-based vaccines to cancer patients. Reverse genetics technology allowed developing NDV into a vector suitable for gene therapy. Examples will be provided in which genetically engineered NDV is being used successfully as vector against new emerging viruses.

## 1. Introduction

Studies on the activities of RNA in the cell, in particular about RNA interference against viruses [1,2], have revolutionized our understanding of the many roles played by this macromolecule. The discovery that RNA can not only replicate but also possess catalytic activity suggested that a single macromolecule could have originally carried out both replication of information and catalysis. Viral sequences in the genomes of various organisms and unexpected amounts of viruses and phages in the biosphere suggest that viruses helped in building genomes and in driving evolution [3].

This article will deal with the contrasting effects that the avian paramyxovirus *newcastle disease virus* (NDV) exerts in different species like birds, mice and humans [4]. It tries to answer the question how it is possible that a virus which is pathogenic in birds can have beneficial effects in mouse tumors and in cancer patients.

The recognition of 5′-triphosphate (non-capped) viral leader RNA in the cytoplasm of NDV infected human cells by retinoic-acid-inducible gene I (RIG-I) [5] initiates a strong type I interferon (IFN) response [6] and is a good example of RNA–protein interaction. Another example is a recently identified host cell-derived microRNA which targets RIG-I mRNA for degradation [7]. Escape mechanisms from type I interferon responses will be described which are a prerequisite for NDV replication in avian cells and for *ebola virus* (EBOV) replication in human cells.

The review will give an overview of the characteristics and molecular biology of NDV and will then describe its interesting immunobiological aspects, including oncolysis and immunogenic cell death (ICD). A main part will summarize 50 years of clinical application of NDV in cancer patients including successful virus production according to good manufacturing practice (GMP). Reverse genetics of NDV will be dealt with to provide a molecular basis for genetic engineering. Examples of newly engineered recombinant NDV viral vectors to protect against new emerging virus diseases will be given.

## 2. Evolution, Diagnostics and Molecular Biology of *Newcastle Disease Virus* (NDV)

### 2.1. Evolution

During approximately 200 million years of evolution, viruses from mammals (derived from *therapsids*) have had time to adapt to the immune system of their host. This will be exemplified later by EBOV, a devastating pathogen in man. A majority of bird species developed approximately 66 million years ago according to a recent whole-genome analysis [8]. Thus, in comparison to mammals, bird viruses appear to have had a shorter period to adapt to the bird immune system.

### 2.2. History and Classification

NDV is an *avian paramyxovirus type I* (APMV-1) which causes a serious disease in chickens and other birds known as ND [9]. NDV outbreaks were first reported in Indonesia, and, subsequently, in Newcastle-upon-Tyne (UK) in the year 1926. There have been several panzootics of this disease in poultry and in domestic pigeons during the last four decades [10,11]. Efforts in controlling the disease involved the development of new vaccines and vaccination protocols [12].

Since the 1970s, NDV has been considered a useful laboratory virus for replication and virulence studies, as recently summarized [13].

NDV strains are classified as *velogenic*, *mesogenic* and *lentogenic* according to their pathotypes and virulence. Velogenic strains are highly infectious in birds. There are *viscerotropic* and *neurotropic* pathotypes. Once introduced into an avian population, NDV is rapidly transmitted among susceptible birds by either inhalation or ingestion. The virus spreads rapidly between premises by the movement of apparently healthy but infected birds, by movement of people and contaminated equipment, food and water, and by airborne spread from one premise to another. The generalized signs of ND include depression, loss of appetite, abnormal thirst, severe dehydration, emaciation and fever. Mortality can reach up to 100% [9,13].

Mesogenic strains of NDV usually cause respiratory disease in adult chickens while lentogenic strains are not pathogenic. The first contact of the virus in permissive hosts occurs with respiratory epithelial cells. Viral attachment is mediated by the hemagglutinin-neuraminidase protein (HN) protein. This binds to gangliosides and *N*-glycoproteins containing a distinct structure of sialic acid and sugars. Virus to cell binding is followed by the activation of the viral fusion protein F. Fusion of the viral and the host cell membrane [14] then allows the transfer of the viral genome into the host cell`s cytoplasm. There, the 15 kb non-segmented negative single-stranded RNA (ssRNA) is transcribed into mRNAs and is translated into viral proteins [15,16].

### 2.3. Virus Propagation, Diagnostics and Inactivation

Embryonated chicken eggs (ECEs) are universally used for virus isolation. The presence of the virus in the allantoic fluid is determined by the hemagglutination (HA) test. The hemagglutination inhibition (HI) test is commonly used to identify NDV. In addition, panels of monoclonal antibodies can be used to characterize and group isolates.

Three in vivo tests have been used for assessment of the pathogenicity of NDV isolates. These are performed either in nine-day-old eggs, in one-day-old chicks or in six-week-old chickens. Currently, the World Organization for Animal Health uses two parameters to differentiate between virulent isolate (notifiable disease) and avirulent isolate (not notifiable). Since the conventional in vivo pathogenicity tests are time consuming and expensive, several rapid phenotyping methods based on reverse transcription (RT)-PCR have been developed. Of these assays the real-time RT-PCR (RRT-PCR) offers the highest sensitivity [16,17].

NDV is very sensitive to heat (10 to 20 min at 56 °C), lipid solvents, ionic and nonionic detergents, formaldehyde and oxidizing agents. Due to the RNA nature of its genome, the virus is very sensitive to UV irradiation and resistant to γ-irradiation.

### 2.4. Morphology

NDV virions are 100 nm or more in diameter, pleomorphic, but mostly spherical in shape. The virion is enveloped with a lipid membrane from the host cell plasma membrane. The envelope contains two transmembrane glycoproteins—the HN and the fusion (F) protein. These proteins are present as homo-oligomers and form spike-like projections of 8 nm length on the outer surface of the envelope. Beneath the envelope lies a non-glycosylated membrane protein called matrix (M) protein. Inside the virion particle rests the nucleocapsid, which has the classical herringbone morphology with a diameter of 18 nm. The viral *nucleocapsid* consists of a single species of viral RNA (15,186 to 15,198 nucleotides (nt) in length) and replicon complex proteins—the nucleocapsid (N), the phosphoprotein (P) and the large polymerase protein (L) [16].

### 2.5. Virus Production from Cell Culture According to Good Manufacturing Practice (GMP)

NDV can be produced not only from ECEs but also from cell culture. Often it has been produced from human tumor cell lines [18]. For *good manufacturing practice* (GMP) production it is advisable to use avian embryonic established cell lines. Following standard operating procedures (SOPs) under validated aseptic operation in a grade B area, the first step consists of creating a Master Cell Bank (MCB). This is followed by establishing a Master Seed Virus (MSV) and a Virus Seed Stock (MVSS). Qualification of the MSV involves titration via determination of tissue culture infective activity (TCID50) [19], identification by sequencing, and proof for absence of adventitious non viral or viral agents. The next step consists of establishing an NDV manufacturing process in a bioreactor involving upstream and downstream processing. A batch is certified and released only when all batch specification and release criteria are fulfilled. All this has been achieved worldwide for the first time at the IOZK in Germany.

### 2.6. Molecular Biology

The complete genome sequences for many strains of NDV are available on the web at www.ncbi.nlm.nih.gov. Based on complete genome sequences, a phylogenetic tree has been designed [11,16]. The codon-associated rate of change for the six NDV proteins revealed that the highest rate of change occurred at the fusion protein [20].

All genome sizes of NDV are consistent with the “rule of six” that is characteristic of APMV-1 [21]. This rule is thought to arise from the need to fully encapsidate the entire length of the genome with a chain of N protein monomers that span exactly 6 nt. This prevents digestion by RNA nucleases. The genomic RNA contains a 55 nt 3′-extragenic region known as the *leader* and a 114 nt 5′-extragenic region known as the *trailer*. The leader and trailer are the control regions essential not only for transcription and replication, but also for encapsidation of newly synthesized RNAs into virus particles. They flank the six genes (3′-N-P/V-M-F-HN-L-5′) of the viral genome. About 90% of the NDV genome carries coding sequences.

Transcription begins at a single promoter that is present in the leader region. First, a leader RNA of 55 nt is synthesized, which is followed by sequential transcription of the genes in the 3′–5′ order to yield individual mRNAs by stop-start mechanism guided by conserved gene-start and gene-end signals. *Gene-start* initiates synthesis of the mRNA and *gene-end* directs polyadenylation and termination of the mRNA. The leader RNA is neither capped nor polyadenylated and is not functional as mRNA. The mRNAs are capped, methylated and polyadenylated by the viral polymerase [11,16].

The incorporation of two G nucleotides at the *RNA editing site* of the P protein results in the frameshift variant protein V. V is an accessory protein that is involved in antagonizing the avian cellular antiviral response to infection. V interacts in a species-specific fashion with bird cell proteins to inhibit IFN signaling. This leads to degradation of “Signal Transducer and Activator of Transcription 1” (STAT1) and to inhibition of interferon-β (IFN-β) induction [22].

### 2.7. Reverse Genetics

This is a new method by which infectious viruses can be generated entirely from cloned DNA (c-DNA). The ability to recover negative-strand RNA viruses, including NDV, from c-DNA clones has allowed investigating structure and function of individual viral genes and their proteins. Reverse genetics technology has also enabled the design of recombinant live NDV vaccines and vaccine vectors. A suitable cell line is transfected with a plasmid expressing the full-length antigenomic RNA. This is cotransfected with three support plasmids which encode the viral N, P and L under the control of a bacteriophage T7 RNA polymerase promoter. The recovery of recombinant NDV requires activity of the T7 RNA polymerase. This enzyme can be provided by a recombinant *vaccinia virus* expressing the T7 gene or by a cell line which constitutively expresses this polymerase [23]. Further details are contained in two recent reviews [24,25].

## 3. Immunobiology

### 3.1. Tumor Selective Replication

The replication life cycle of all RNA viruses involves the formation of double-stranded RNA (dsRNA). This foreign structure activates an important cellular defence system based on type I interferons, such as IFN-α and -β. Mutations in tumor cells often cripple the interferon system which allows un-inhibited cell proliferation and provides relative resistance to apoptosis [26]. In non-permissive hosts, such mutations make tumor cells to a relatively permissive substrate for the replication of RNA viruses such as NDV. Identified mechanisms that can explain tumor selectivity of viral replication and oncolysis in non-permissive hosts involve: (i) defects in activation of anti-viral signaling pathways [27]; (ii) defects in type I IFN signaling pathways [28]; (iii) defects in apoptotic pathways [29]; and (iv) activation of Ras signaling and expression of Rac1 protein [30].

Infection of cells by NDV involves basically two steps: (1) cell binding, membrane fusion [14], transduction of the viral genome and transcription of viral genes; and (2) viral replication using a newly produced nucleocapsid as anti-genome [25,31]. Infection of normal cells from non-permissive hosts usually does not proceed to the second step [25,26,27,32]. In contrast, in nearly all tested human/murine tumor cells and transformed cells, NDV infection proceeded to the second step thus allowing tumor selective replication [33]. NDV has the ability to replicate up to 10,000-fold faster in human cancer cells than in most normal human cells [34].

### 3.2. Oncolysis and Immunogenic Cell Death

NDV has a number of desirable properties that make it an attractive *oncolytic* agent in non-permissive (not avian) hosts such as mouse or man. It is known to enter cells by binding to sialic acid residues, which are present on most human cancer cells, making it suitable for use in a broad range of cancer cell types. NDV is a fast growing virus and is highly fusogenic in tumor cells. There is virtually no pre-existing immunity to NDV in the general human population.

An important new paradigm of oncolytic virus-mediated immunotherapy is the concept of *immunogenic cell death* (ICD) [35]. Classical physiological apoptosis is non-immunogenic. The features of ICD induced by NDV in tumor cells include an endoplasmatic reticulum (ER) stress response, immunogenic apoptosis, necrosis and autophagy [36]. Such ICD features lead to shutdown of protein synthesis, surface exposure of calreticulin, heat-shock proteins (HSPs), and of the viral proteins HN and F. Upregulation of major histocompatibility class I protein (MHC I) and adhesion molecules (ICAM-1 and LFA-3) lead to improved antigen presentation [37]. *Immunogenic apoptosis* selectively recruits neutrophils as first innate immune responders. Infection by NDV causes release of the neutrophil attracting chemokines CXCL1, CCL2 and CXCL10 [38]. Neutrophil recruitment by chemokines seems an old evolutionary conserved mechanism across vertebrates since chemokines evolved about 650 million years ago in fish [39].

Pathogen-associated molecular patterns (PAMPs) from NDV recognized by respective pattern recognition receptors (PRRs) from innate immunity cells are: (i) 5′-triphosphate viral leader RNA [5]; (ii) dsRNA [30]; and (iii) the HN protein [40,41]. These PAMPs are recognized, respectively, by the following PRRs: (i) cytoplasmic RIG-I; (ii) cytoplasmic dsRNA dependent protein kinase R (PKR) and endosomal Toll-like receptors (TLRs); and (iii) plasma membrane expressed NK cell receptor NKp46 [41]. PRRs initiate multiple response pathways leading to a strong type I interferon response, to release of pro-inflammatory cytokines and to activation of immune cells. Finally, cell necrosis leads to release of damage-associated molecular patterns (DAMPs), and of further cytokines and chemokines [37].

### 3.3. Post-Oncolytic Immune Response

In an orthotopic murine glioma model it was demonstrated that NDV virotherapy induces ICD with its molecular determinants such as calreticulin, HSP and high mobility group box-1 (HMGB1, amphoterin). This is followed by tumor-specific immune T cell memory [42].

### 3.4. Immunostimulatory Properties

The viral surface protein HN was shown to be recognized by the *PRR Nkp46* of murine NK cells and to transmit cytotoxicity inducing signals [41]. Transfected and cell surface exposed HN but not F molecules induced in a paracrine stimulation assay in human peripheral blood mononuclear cells (PBMC) cell surface expression of *TNF-related apoptosis-inducing ligand* (TRAIL) and secretion of IFN-α [39]. Upon contact with NDV, not only NK cells but also monocytes become activated to produce tumoricidal activity through TRAIL [43]. NDV infected macrophages produce nitric oxide (NO) via activation of nuclear factor-kappa B [44].

The effect of NDV on human dendritic cells (DCs) has been studied in a sophisticated way using technologies developed by systems biology [45]. Due to its avian origin, NDV was considered a prototype virus to study an uninhibited cellular response with human DCs. The anti-viral state transition of the DCs occurred within 18 h post-infection by NDV. The change of the genetic program was explained by a regulatory network of 24 distinct transcription factors (TFs). These regulated the expression of 779 of the 1351 up-regulated genes. The anti-viral state transition was associated with polarization of the DCs towards type 1 (DC1). This type of polarization favors the induction of T helper type 1 (Th1) T cell responses upon antigen presentation by the DCs.

The genetic re-programming of DCs by NDV justifies the term “priming” which is usually associated only with cells of the adaptive immune response (T and B lymphocytes).

With regard to T cells, HN molecules at the surface of infected tumor cells were shown to introduce new cell adhesive strength for interaction with lymphocytes [46] and for T-cell costimulation, including CD4+ T helper [47] and CD8+ cytotoxic T cells [48].

### 3.5. Type I Interferon Response

Type I IFNs exert their antitumor effects either directly, by targeting the tumor cells or the tumor stem cells. Antitumor effects are achieved also indirectly, by regulating the anticancer activities of the immune system. IFN-β and IFN-α exhibit antiproliferative effects by p53 induction, CD8+ T lymphocyte and macrophage activation, chemokine secretion and miR-21 downregulation [48].

#### 3.5.1. Studies with Human Cells

NDV induces in human PBMC, DCs and monocytes a strong type I IFN response [4,6,15,40]. This occurs within 18–24 h and consists of an early phase and a late phase. The early phase is initiated through the recognition of PAMPS through PRRs (see Section 3.2). A signal transduction cascade to the nucleus then induces IFN-β first followed by IFN-α. During the late phase of the IFN response, the induced and secreted IFN-β and -α molecules interact with the cell surface expressed α-chain of the *type I IFN receptor* (IFNRA). This initiates an amplification loop of the IFN response involving STAT proteins and the interferon regulatory factor IRF-9.

#### 3.5.2. Studies with Murine Cells

In murine studies an association was described between the strength of the type I interferon response and the resistance of cells (normal or neoplastic) to virus replication following infection by NDV. The studies revealed an important role in the IFN response of RIG-I, IRF3, IFN-β and IRF7 [6,27]. In addition, studies with cells from Knock-out mice devoid of the gene for IFNRA showed the importance of this receptor: In its absence, normal cells were incapable of preventing NDV replication [49,50].

Type I interferon was demonstrated to be important for the generation of cytotoxic T lymphocyte (CTL) responses. In vivo, a low basic level of IFN is constitutionally expressed. Following application of a neutralizing anti-IFN antibody in mice, it was impossible to induce in these CTL precursor cells. In addition, in vitro, in CTL restimulation cultures, the addition of a neutralizing anti-IFN antibody inhibited the CTL response. The augmented tumor-specific CTL response observed in mice immunized with NDV infected tumor cells in comparison to mice immunized with non-infected tumor cells was mediated via IFN-α/β [51] and was a result of CD4+ and CD8+ immune T cell cooperation [52].

Intranasal application of NDV in mice induced in lung epithelial cells pro-inflammatory cytokines and type I interferon which is important also for counter-acting *regulatory T cell* (Treg) activity [49].

### 3.6. Immune Escape from Type I Interferon Responses

Immune escape from type I interferon responses in permissive NDV infected avian cells is mediated via the V protein, a frameshift variant of the viral phosphoprotein P. V inhibits IFN signaling by targeting STAT1 for degradation [44,53]. It also inhibits IFN-β induction through interaction with melanoma differentiation-associated gene 5 (MDA5), leading to the inhibition of IRF3 activation [54]. The V protein shows specificity for avian proteins and does not interfere with the IFN response in mammalian cells [55].

Ebola virus (EBOV) from primates has during evolution developed immune escape mechanisms from the type I IFN response of primate cells [50]. EBOV, first discovered in 1976, belongs to the largest known RNA viruses. With a diameter of 80 nm and a length of up to 14,000 nm it is a member of the *Filoviridae* family. Similar to NDV, the genome of EBOV consists of a negative ssRNA. It codes for 8 proteins. Two of the eight viral proteins, Vp24 and Vp35, are involved in immunosuppression. Vp35 antagonises the early phase of the interferon response.It binds directly to dsRNA and inhibits anti-viral signaling. It also blocks virus induced IRF3 phosphorylation. This prevents IRF3 dimerization and nuclear translocation. In cells expressing Vp35, there was also an observed suppression of phosphorylation of PKR and of elongation initiation factor 2a (eIF-2a) [56]. In contrast, Vp24 is an antagonist of the late phase. Vp24 binds directly to STAT1 and inhibits its nuclear translocation [57].

Table 1 summarizes the described molecular determinants for RNA viral immune stimulation (NDV in man) and for RNA viral immune escape (NDV in birds and Ebola in man).

### 3.7. Anti-Tumor Effects in Vivo

Intra-tumoral and intra-peritoneal injection of oncolytic NDV (mesogenic attenuated strain *PV701*) caused durable, complete tumor regression in athymic mice bearing human neuroblastoma and fibrosarcoma xenografts [60]. Recombinant NDV strains derived from the velogenic strain *italien*, after intra-tumoral injection, induced syncytium formation and cell death. It also prolonged the survival of tumor-bearing athymic mice and suppressed loss of body weight [61].

Orthotopic glioma studies in immunocompetent syngeneic mice revealed that intra-tumoral virotherapy with NDV induced through ICD long-term survival [42]. The induced protective immunity was dependent on a functional adaptive immune system and on CD8+ T cells [42].

These latter recent results corroborate earlier findings from the 1980s of post-operative anti-tumor vaccination in the murine ESb lymphoma model with an autologous NDV infected tumor cell vaccine. The induced immunity included CD4+ Th and CD8+ CTL [51,52], was highly specific, protected against outgrowth of metastases and increased significantly the percentage of long-term surviving mice [62].

Anti-tumor effects of NDV can also be expected in other non-avian vertebrate species. This should be the case with tumors from domestic animals such as cat and dog and from animals of veterinary medicine, like horse, pig and cow, another area of future NDV application.

### 3.8. Potential to Break Therapy Resistancies

NDV has the capacity to replicate in non-proliferating tumor cells, such as X-irradiated tumor vaccine cells. Since virus replication in the cytoplasm is independent of cell proliferation, oncolytic NDV has the potential to target tumor stem cells or cells from tumor dormancy, both of which may not be affected by chemo- or radiotherapy [4,63].

Furthermore, NDV can replicate in apoptosis-resistant tumor cells [29], in hypoxic cancer cells [64] and in interferon-resistant tumor cells [6,23,27,28].

Resistance to immunotherapy can also be broken by NDV infection. Human T cell tolerance to melanoma could by broken by melanoma infection with NDV (strain *Ulster*) [47]. In a mouse melanoma model, localized oncolytic NDV virotherapy was demonstrated to overcome systemic tumor resistance to immune checkpoint blockade immunotherapy [65]. This is important and suggests that results from clinical application of immune checkpoint blockade immunotherapy can be improved by combination with oncolytic NDV.

### 3.9. Anti-Fibrotic Activity

Liver fibrosis is a major health problem and the 12th most common cause of death in the Unites States [66]. Activated *hepatic stellate cells* (HeSCs) are the crucial factor responsible for liver fibrosis. HeSCs are liver-resident bone marrow derived mesenchymal stem cells located in the space of Disse and store up to 80% of total body vitamin A. Upon activation, the star shaped HeSCs differentiate into myofibroblasts to produce extracellular matrix (ECM), thus contributing to liver fibrosis. When NDV was injected to hepatic fibrosis mice, regression of liver fibrosis was observed. Replication of NDV in murine primary HeSCs or in human LX-2 HeSC cells was demonstrated by colocalization of NDV and α-smooth muscle actin (α-SMA). Virus infection reduced the production of collagen fibrils and of matrix metalloproteinase (MMP). Virus replication in HeSCs was followed by cell death via apoptosis [67]. Considering the importance of liver fibrosis and its connection to *hepatocellular carcinoma* (HCC), these findings have a strong potential clinical impact.

## 4. Industrial Application

A number of NDV strains have been used for preventive vaccination against ND in the poultry industry [12,16]. Vaccination is performed using either live or inactivated vaccines [68]. Mass application of live vaccines by sprays and aerosols is very popular. Several lentogenic strains, such as *LaSota* and *B1* have been used for more than 50 years with proven track record of safety and efficacy. However, they do not completely prevent infection or virus shedding [68]. They also do not possess genetic markers to allow differentiation between infected and vaccinated birds. Recently, a recombinant ND vaccine “*Innovax-ND*” was approved by the US FDA for commercial use [16]. It was recently also shown that an attenuated genotype VII NDV vaccine generated by reverse genetics effectively protected the vaccinated birds against homologous virulent genotype VII virus challenge and reduced virus shedding significantly more than in *LaSota* vaccinated animals [69].

Although it is not known which arm of the immune response nor which viral envelope glycoprotein is more important in providing protection against ND, it is known that a robust immune response is required to completely cease virus replication and provide long-term protection. This can likely only be induced by both envelope glycoproteins and participation of innate and adaptive cell mediated and humoral immune responses [16].

## 5. NDV as Therapeutic in Cancer Patients

### 5.1. High Safety Profile

Certain types of viruses have oncolytic and/or other anti-neoplastic properties and can be used for virotherapy in cancer patients. Advantages exist in using oncolytic paramyxoviruses in comparison to viruses from other families (e.g., retroviruses or DNA viruses). There is a lack of gene exchange via recombination and a lack of interaction with host cell DNA. Even relatively high doses of NDV are well tolerated by cancer patients. The reported side effects are mild (grade 1 and 2), with the most common being mild fever [4,15].

### 5.2. Treatment by Oncolytic NDV

In the 1990s, a placebo-controlled Phase II clinical trial performed by Csatary in Hungary [70] included 33 late-stage cancer patients in the NDV treatment group and 26 in the placebo group. They used an attenuated oncolytic veterinary vaccine strain (*Mukteswar*) which they termed *MTH-68/H*.

The virus was applied via inhalation to target lung metastases. Four thousand infectious units/day of NDV was inhaled twice weekly for six months. The clinical results suggested a decrease in cancer-related symptoms and better survival [70]. This was, however, disputed because the study was not randomized and consisted of very heterogeneous groups of patients. Csatarys group later reported about a selected case series study of four high-grade gliomas treated with *MTH-68/H*. This study showed radiographically-documented responses and long survival with improved symptomatology [71].

Another oncolytic NDV strain (*PV701*) was tested in patients with advanced cancers in a Phase I trial conducted in the United States [72].

The patients (*n* = 79) were given escalating doses of virus intravenously. Their tumors had not responded to conventional therapy. Doses of three billion infectious particles were well tolerated. Dose-limiting toxicities included dyspnea, dehydration and diarrhea. When patients were desensitized with a lower initial dose, the maximal tolerated dose was increased 10-fold [73]. This suggests a “priming” or pre-conditioning effect on the immune system (see Section 3.4 and Section 5.6).

### 5.3. Treatment by NDV Oncolysate Vaccines

Cassel from Atlanta (GA, USA) reported as early as 1965 on NDV (oncolytic strain *73 T*) as an antineoplastic agent [74]. When they observed the development of post-oncolytic anti-tumor immunity [75], they focused on the development of viral oncolysates as vaccines. They used such vaccines for post-operative management of malignant melanoma patients. Vaccinations were performed over many years and were never stopped completely. Details about 2 Phase II clinical studies involving 32 and 51 patients with Stage II metastatic melanoma (AJCC stage III) have been summarized [8]. Cassel concluded from his results: “The unusual disease-free survival periods, including exceptional survivals in 21 patients with head and neck disease and six cases with cerebral metastases, suggest a unique role for the administration of NDV oncolysate in the management of Stage II melanoma patients” [76].

### 5.4. Treatment by Autologous Tumor Cell Vaccine modified by infection with NDV (ATV-NDV)

My group at the German Cancer Research Center (DKFZ) in Heidelberg, had established in the 1980s an animal model system to study cancer metastasis and immunotherapy. Our concept was to use a live cell NDV infected tumor cell vaccine rather than a oncolysate because live cell vaccines apparently have higher immunogenicity than lysates. The vaccine was termed *ATV-NDV*. It consisted of 10 million irradiated tumor cells, which had been infected with NDV (lentogenic strain *Ulster*). The anti-metastatic effects observed in the pre-clinical studies, the specificity of the protective immunity induced and the mechanism of function have been summarized elsewhere [4,62,63].

Translational research from mouse to man was performed in the 1990s. After a series of tests of human tumor cell lines or of tumor cells from fresh operation specimens we developed a protocol to produce a human ATV-NDV vaccine which had pleiotropic immune stimulatory properties [31]. With this type of vaccine, more than 10 Phase I to II clinical post-operative vaccination studies were performed in patients with different types of cancer in the years from 1990 to 2008 [4,63,77]. The trials involved, among others, carcinomas of breast [78], colon, rectum [79], kidney [80], head and neck [81], pancreas [77] and glioblastoma multiforme (GBM) [82]. Only two studies will be summarized.

(i) Patients suffering from GBM (*n* = 23) were vaccinated with ATV-NDV from cell culture to assess feasibility, safety and clinical benefit in a Phase I study [82]. The median progression-free survival (PFS) of vaccinated patients was 40 weeks (vs. 26 weeks in 87 non-vaccinated control subjects from the same time period and the same clinic). The median overall survival (OS) was 100 weeks (vs. 49 weeks in control subjects, *p* < 0.001). In the vaccinate group, immune monitoring revealed significant increases of skin anti-tumor delayed-type hypersensitivity (DTH) reactivity, of the number of tumor-reactive memory T cells (MTCs) in the blood and in the numbers of CD8+ tumor-infiltrating T lymphocytes (TIL) in frozen tissue slices from GBM recurrences. There was one complete remission of non-resectable tumor mass [82];
(ii) A prospectively randomized Phase II/III trial investigated the efficiency of ATV-NDV after liver resection for hepatic metastases of colorectal carcinoma (CRC) as a tertiary prevention method [83]. Stage IV CRC patients (*n* = 25) were vaccinated and compared with a similar number of non-vaccinated comparable patients for an exceptionally long follow-up period of about ten years. At the end, there was no significant difference between the vaccinated and the control arm. However, when stratified for tumor localization, there were clear-cut differences between colon and rectum. A significant benefit was seen in the colon but not in the rectum cancer subgroup in terms of long-term metastasis-free survival and OS. In the colon cancer control arm, 78.6% had died, in the vaccinated arm only 30.8% [83].

The latter trial corroborated similar results (about 30% increase in OS) obtained in most of the other studies [4,15,33,63]. Since the latter study was performed as a prospectively randomized Phase II/III study, it provides clinical evidence for the effectivity and potential of the cancer vaccine ATV-NDV.

The potential mechanism of function of the long-term survival observed in stage IV colon carcinoma patients has been described in detail [59]. An important role in long-term survival can be associated with cancer-reactive MTCs. These can be “primed” against tumor-associated antigens (TAAs) in cancer patients either spontaneously or upon anti-tumor vaccination. Bone marrow has been demonstrated as a priming site for T-cell responses to blood-borne TAAs [84]. Patient-derived re-activated MTCs from bone marrow, upon transfer to NOD/SCID mice bearing autologous human tumor xenotransplants, were capable of tumor rejection [85], thus demonstrating their importance.

Table 2 summarizes the various mentioned anti-tumor effects of NDV in mouse and man.

### 5.5. Treatment by ATV-NDV with Attached Bispecific Antibody

In spite of these promising results, we generated new ideas for further improvements. This appears necessary considering the fact that there is still a majority of patients that has to be considered as non-responders to this immunotherapy with NDV-based tumor vaccines.

One strategy of improvement consisted in the augmentation of T-cell costimulatory signals. This can be achieved by the attachment to ATV-NDV of NDV-specific single chain antibodies with dual specificity (bispecific (bs) antibody fusion proteins), for instance anti-HNxanti-CD28 (bsHN-CD28) [87]. CRC patients with late-stage disease that could no longer be subjected to surgery with curative intent (*n* = 14) were treated with *ATV-NDV-bsHN-CD28*. No severe adverse events were noticed in this Phase I trial. Before vaccination none of the patients had detectable levels of tumor-reactive blood circulatory MTCs. All patients showed an immunological response of tumor-reactive MTCs, at least once during the course of five vaccinations. Additionally, there was a dose-response relationship with the costimulatory molecule attached to the vaccine. A partial response of metastases (>30% decrease, RECIST criteria) was documented in four patients [87].

### 5.6. Treatment by Autologous NDV Oncolysate-Pulsed DCs

The other strategy for improvement was to combine viral oncolysate (VOL) from ATV-NDV with dendritic cells (DCs). VOL-pulsed DCs (VOL-DCs) were developed at the Immunological and Oncological Center (IOZK) in Cologne (Germany). The idea was meant to improve the de novo generation of TAA-specific cells from naïve T cells. In 2015, IOZK received an official permit for an individual application of this Advanced Therapeutic Medicinal Product (ATMP) to cancer patients.

The new strategy of cancer immunotherapy at IOZK consists in combining hyperthermia and oncolytic virus pretreatment with specific autologous anti-tumor vaccination [86]. Hyperthermia can activate the immune system when it is adjusted to reach a tissue temperature of 38.5 to 40.5 °C. Systemic oncolytic NDV virus pretreatment has a positive “pre-conditioning” effect (see Section 3.4 and Section 5.2) on the patient’s immune system and induces VOL-reactive CD4+ T helper cells [86]. These create systemic recall responses at the site of VOL-DC vaccination, induce the chemokine CCL3 and thereby enhance DC migration to the draining lymph nodes with improved antitumor efficacy [88]. In vitro, CD4+ T helper cells increased the maturation of human DCs much better than the maturation stimuli CD40-L and IFN-γ [89].

One case-report from IOZK demonstrates long-term remission of prostate cancer in a patient with extensive bone metastases. The patient had failed standard therapy. The above treatment induced a long-lasting tumor-reactive MTC response [90]. Another case report relates to long-term survival of a breast cancer patient with extensive liver metastases. After operation the patient refused further standard therapy. Instead, she was treated with the above strategy at IOZK. The patient survived more than 66 months after the initial diagnosis. In addition, a continuous high quality of life was reported [91].

New results from IOZK relate to case series studies of GBM patients [86]. Kaplan-Meier survival analysis of 10 newly diagnosed operated patients show a median Overall Survival (OS) of 30 months. This has to be compared to 14.6 months after standard radio/chemotherapy according to the Stupp protocol. Remarkable is also a 5-year survival in the current series with combinatorial immunotherapy of almost 20% [86].

### 5.7. Future Aspects: Harnessing Oncolytic Virus-Mediated Anti-Tumor Immunity

This research topic is addressed in a recent e-book from Frontiers in Oncology [92]. How can oncolytic viruses (OVs) be harnessed or combined with other agents to mediate stronger anti-tumor effects? Among others, the following strategies were proposed and discussed: (i) combine OVs with immune checkpoint blocking antibodies [93]; (ii) combine OVs with hyperthermia [86]; (iii) combine OVs with activated T cells [94]; (iv) combine OVs with approved drugs and novel small molecules to dampen the innate and adaptive anti-viral responses, increase the anti-tumor immune response, or both [95]; (v) combine OVs with chemotherapy or pharmaceutical immunomodulators [96]; snf (vi) improve tumor targeting. The specificity and efficacy of systemic tumor targeting of NDV could be improved using a bispecific adapter protein. This also reduced unwanted side-effects [97]. Tumor targeting of grafted T cells could become improved via cell-bound tri-specific antibodies directed against a tumor-introduced viral antigen such as HN of NDV [94].

## 6. NDV as Vector against New Emerging Virus Diseases

### 6.1. Viral HN Gene as Vaccine Adjuvant

Application of a plasmid encoding the HN protein of NDV (pHN) at the ear pinna of mice induced high levels of systemic IFN-α and reduced tumor growth. In tumor bearing mice, vaccination with pHN DNA compared to a control DNA caused a significant increase in NK cell infiltration and a decrease in infiltration of *myeloid-derived suppressor cells* (MDSCs) [58].

### 6.2. Recombinant NDV as a Vaccine Vector

Recent studies underline the potential of recombinant NDV (recNDV) as a vaccine vector. The expression levels of foreign proteins, such as *influenza virus* hemagglutinin [98] are high and the foreign genes are very stable after many virus passages in vitro and in vivo. RecNDV can be engineered even with two genes to secrete full intact antibodies if respective heavy and light chains of IgG from a hybridoma are incorporated into the viral genome [98].

### 6.3. RecNDV as a Vaccine Vector against Emerging Pathogens

New viruses or variants can cause epidemics and pose huge public health problems and economic losses within a short time.

NDV-based live attenuated vaccine completely protected chickens and mice from lethal challenge with homologous and heterologous *H5N1 avian influenza viruses* [99]. Similar results have been obtained with recNDV engineered to express the spike *S. glycoprotein* of *severe acute respiratory syndrome coronavirus* (SARS-CoV). After intranasal immunization, this vaccine protected African green monkeys against challenge with a high dose of SARS-CoV virus [100]. NDV seems particularly suited as a vector against respiratory viral infections.

NDV may be employed as an agent for immune stimulation and for prophylactic conditioning of the host immune system against the risk of viral infection (see Section 3.4, Section 5.2 and Section 5.6). This may be particularly relevant for individuals who come into contact with patients infected for instance with EBOV [50]. A recNDV engineered to express EBOV viral antigens could be well suited as a prophylactic vaccine because it would express HN as adjuvant to stimulate a strong type I interferon response as well as target antigens from EBOV. Virus-like particles, which also activate type I interferon pathways, were shown to facilitate post-exposure protection against EBOV [101].

*Acanthamoeba polyphaga Mimivirus* (APMV) was isolated from *amoebae* in a hospital while investigating a *pneumonia* outbreak. A study of interaction of APMV with human cells revealed that the virus is able to evade the IFN system by inhibiting the regulation of interferon-stimulated genes, suggesting that the virus and humans had host–pathogen interactions [102]. This is another example where a recNDV vector might in future become very usefull.

Table 3 contains future strategies for harnessing oncolytic virus-mediated anti-tumor immunity and for using NDV as vector against new emerging virus diseases.

## 7. Conclusions

There is much that can be learned from an avian RNA virus such as NDV. The virus was capable, during evolution, of adapting to the immune system of birds by generating the frameshift variant protein V that interferes with the type I interferon response of cells from birds. Emerging viruses causing fatal diseases in man such as Ebola developed similar viral interferon escape mechanisms. Reverse genetics technology enables the development of NDV into a vector for incorporation of foreign genes. Such vectors are capable of protecting, for instance, chickens against H5N1 avian influenza virus and African green monkeys against SARS-CoV virus.

Tumor cells, by somatic mutation, often develop mechanisms to escape the growth inhibiting effects of type I interferons. In non-permissive hosts, such as mouse and human, this allows the avian virus NDV to replicate selectively in tumor cells and to exert in these an oncolytic effect. Regarding medical application of NDV in cancer patients, the following immunobiological mechanisms are important: Oncolytic NDV destroys tumor cells and induces a cell death that is immunogenic and initiates a DC1 and Th1 directed anti-tumoral T cell response. This leads to further destruction of tumor cells and to development of tumor-reactive memory T cells. The latter play an important role for long-term survival. In addition, NDV has the potential to break various types of therapy resistances. These findings explain the promising effects seen with oncolytic NDV and NDV-based vaccines (e.g., ATV-NDV, VOL-DC) in cancer patients. The IOZK in Cologne (Köln, Germany) is the first and only place where VOL-DC as an ATMP is being applied with official permit. If in future NDV would become included into protocols for standard treatment, many more cancer patients would be able to profit from this than hitherto.

NDV fulfills many prerequisites for a new therapeutic drug in man: high safety profile, low side effects in comparison to chemo- or radiotherapy, many-fold anti-neoplastic effects, strong type I IFN response and broad immunostimulatory effects. As reviewed here, NDV has various applications: (i) as viral vaccine in poultry; (ii) as recombinant vector against emerging pathogens; and (iii) as OV and immune stimulant in cancer patients. There is good reason to expect many benefits from further research and application of NDV.

## Figures and Tables

**Table 1:** Examples of molecular determinants for RNA viral immune stimulation and for RNA viral immune escape for NDV and Ebola virus.

**1. NDV in Man**	
Viral PAMPs:	5′triphosphate leader RNA [5]
	ssRNA, dsRNA [5,26,32,56]
	HN protein [40,41,46,48,58]
Host cell PRRs:	cytoplasmic RIG-I [5]
	endosomal TLR [35]
	plasma membrane NKp46 [41]
Response:	Inhibition of infection by production of type I IFNs [58]
	Activation of NK cells killing infected cells [41]
	Priming of DC1, Th1, CTLs and MTCs [45,47,48,59]
**2. NDV in Birds**	
Type I interferon response counteracted by the viral V protein [53,54,55]	
V protein targets STAT1 for degradation [53]	
V protein interaction with RIG-I/MDA5 leading to downregulation of IRF3 and IFN-β [54,55]	
**3. Ebola in Man**	
Type I interferon response counteracted by two viral proteins: Vp35 and Vp24 [56,57]	
Vp35 mediated interaction with dsRNA leading to downregulation of IRF3, PKR and eIF-2α [56]	
Vp24 mediated interaction with STAT1 leading to downregulation of its nuclear translocation [57]	

Abbreviations: eIF-2a, Elongation initiation factor 2a; MDA5, Melanoma differentiation-associated gene 5; PAMPs, Pathogen-associated molecular patterns; PRRs, Pattern recognition receptors; RIG-I, Retinoic-acid-inducible gene I; PKR, dsRNA dependent protein kinase R; TLR, Toll-like receptor; HN, Hemagglutinin-neuraminidase protein; IFN-β, Interferon β; IRF3, Interferon response factor 3; ssRNA, single-stranded RNA; dsRNA, double-stranded RNA; IFN, type I interferon; NK, natural killer cell; DC1, Dendritic cell polarized towards type 1; Th1, T helper cell polarized towards type 1; CTL, Cytotoxic T lymphocyte; MTC, Memory T cell; STAT, Signal transducer and activator of transcription.

**Table 2:** Anti-tumor effects of NDV in mouse and man.

**1. Tumors and Fibrosis in Mice**
Orthotopic glioma, primary tumor [42]
Syngeneic lymphoma (ESb), as secondary preventive method against metastases [62]
Xenografted human tumors in athymic mice, primary tumors [60,61]
Syngeneic melanoma (B16), breaking systemic tumor resistance to immune checkpoint blockade [65]
Liver fibrosis, regression [67]
**2. Tumors in Man**
Malignant melanoma, stage II (AJCC stage III), NDV oncolysate vaccine, post-operative (p-o) application [76]
Primary breast carcinoma, ATV-NDV vaccine, p-o application [78]
Primary colorectal carcinoma, ATV-NDV vaccine, p-o application [79]
Advanced renal cell carcinoma, ATV-NDV vaccine + cytokines, p-o application [80]
Primary head and neck squamous cell carcinoma, ATV-NDV, p-o application [81]
Glioblastoma multiforme (GBM), ATV-NDV, p-o application [82]
High grade glioma, oncolytic NDV i.v. [71]
GBM, hyperthermia/NDV pretreatment + VOL-DC vaccination [86]
Advanced colorectal carcinoma, stage IV, ATV-NDV after resection of liver metastases, prospective randomized trial [59,83]

Abbreviations: ATV-NDV, Autologous tumor cell vaccine modified by infection with NDV; GBM, Glioblastoma multiforme; p-o, post-operative; VOL-DC, Dendritic cell vaccine pulsed with vial oncolysate.

**Table 3:** Future aspects.

**1. Harnessing Oncolytic Virus-Mediated Anti-Tumor Immunity**
Combine Oncolytic Viruses (OVs) with immune checkpoint blocking antibodies [65]
Combine OVs with hyperthermia and activated T cells [86,90,91]
Use bispecific and trispecific antibody fusion proteins to improve tumor targeting of T cells [94] and of OVs [97]
Combine OVs with approved chemotherapeutic drugs and small molecules [96]
Combine OVs with pharmaceutical immunomodulators [95]
**2. Using NDV as Vector against New Emerging Virus Diseases**
Use viral HN gene as vaccine adjuvant [103]
Incorporate into a recNDV vector one or more foreign genes to achieve stable and high expression levels [98,99]
Follow positive experiences with avian influenza viruses [100] and SARS-CoV [101]

Abbreviatons: HN, Hemagglutinin-neuraminidase protein; OV, Oncolytic virus; recNDV, recombinant Newcastle disease virus vector; SARS-CoV, Severe acute respiratory syndrome coronavirus.

## Abbreviations

APMV	Acanthamoeba Polyphaga Mimivirus
APMV-1	Avian Paramyxovirus Type 1
ATMP	Advanced Therapeutic Medicinal Product
ATV-NDV	Autologous Tumor Cell Vaccine Modified by Infection with NDV
bs	Bispecific
CD40-L	CD40 Ligand (CD154)
c-DNA	Cloned DNA
CRC	Colorectal Carcinoma
CTL	Cytotoxic T Lymphocyte
DAMP	Damage Associated Molecular Pattern
DC	Dendritic Cell
DKFZ	German Cancer Research Center Heidelberg
dsRNA	Double-Stranded RNA
DTH	Delayed Type Hypersensitivity
EBOV	Ebola Virus
ECE	Embryonated Chicken Egg
ECM	Extracellular Matrix
eIF-2α	Elongation Initiation Factor 2α
GMP	Good Manufacturing Practice
HA	Hemagglutination Assay
HCC	Hepatocellular Carcinoma
HeSC	Hepatic Stellate Cells
HI	Hemagglutination Inhibition
HMGB1	High Mobility Group Box-1 Protein
HN	Hemagglutini-Neuraminidase Protein
HSP	Heat Shock Protein
ICAM-1	Intercellular Adhesion Molecule 1
ICD	Immunogenic Cell Death
IFN	Interferon
IFNRA	Receptor α Chain for Type I IFN
IOZK	Immunological and Oncological Center Cologne
IRF	Interferon Response Factor
LFA-3	Lymphocyte Function Associated Antigen 3
MCB	Master Cell Bank
MDA5	Melanoma Differentiation-Associated Gene 5
MDSC	Myeloid Derived Suppressor Cell
MMP	Matrix Metalloproteinase
mRNA	Messenger RNA
MSV	Master Seed Virus
MVSS	Master Virus Seed Stock
MTC	Memory T Cell
ND	Newcastle Disease
NDV	Newcastle Disease Virus
NK	Natural Killer Cell
NO	Nitric Oxide
nt	Nucleotide
OS	Overall Survival
OV	Oncolytic Virus
PAMP	Pathogen Associated Molecular Pattern
PBMC	Peripheral Blood Mononuclear Cell
PFS	Progression-Free Survival
PKR	dsRNA Dependent Protein Kinase R
p-o	Post-Operative
PRR	Pattern Recognition Receptor
RIG-I	Retinoic-Acid-Inducible Gene I
RNA	Ribonucleic Acid
RT-PCR	Reverse Transcription PCR
RRT-PCR	Real-Time RT-PCR
SARS-CoV	Severe Acute Respiratory Syndrome Coronavirus
SMA	Smooth Muscle Actin
ssRNA	Single-Stranded RNA
STAT	Signal Transducer and Activator of Transcription
SOP	Standard Operating Procedure
TAA	Tumor-Associated Antigen
TF	Transcription Factor
Th1	T Helper Cell (Type 1)
TIL	Tumor Infiltrating Lymphocyte
TLR	Toll-Like Receptor
TRAIL	TNF-Related Apoptosis-Inducing Ligand
V	V Protein of NDV
VOL	Viral Oncolysate
